# Acute Effects of Assisted Cycling Therapy on Post-Stroke Motor Function: A Pilot Study

**DOI:** 10.1155/2019/9028714

**Published:** 2019-02-13

**Authors:** Simon D. Holzapfel, Pamela R. Bosch, Chong D. Lee, Patricia S. Pohl, Monica Szeto, Brittany Heyer, Shannon D. Ringenbach

**Affiliations:** ^1^College of Health Solutions, Arizona State University, USA; ^2^Department of Physical Therapy and Athletic Training, Northern Arizona University, USA; ^3^College of Medicine in Phoenix, University of Arizona, USA

## Abstract

**Background:**

Stroke is the most common cause of long-term disability in the United States (US). Assisted Cycling Therapy (ACT) at cadences of about 80 rpm has been associated with improvements in motor and clinical function in other clinical populations. The acute effects of ACT on motor function of persons with stroke have not been investigated.

**Objectives:**

The primary purpose of this cross-over trial was to compare the effects of ACT, voluntary cycling (VC), and no cycling (NC) on upper (Box and Blocks Test) and lower extremity motor function (Lower Extremity Motor Coordination Test) in adults with chronic stroke (age: 60 ± 16 years; months since stroke: 96 ± 85). The secondary purpose was to examine average cycling cadence and ratings of perceived exertion as predictors of change in motor function following the exercise session.

**Methods:**

Twenty-two participants (female = 6, male = 16) completed one 20-min session each of ACT (mean cadence = 79.5 rpm, VC (mean cadence = 51.5 rpm), and NC on separate days in quasi-counterbalanced fashion).

**Results:**

Main effects of intervention did not differ between ACT and VC. Within-intervention analyses revealed significant (p < 0.05) pre- to posttest changes in all outcome measures for ACT but only in the Lower Extremity Motor Coordination Test on the non-paretic side for VC. Trend analyses revealed a positive relationship between average ACT cadences and improvements in upper and lower extremity motor function (p < 0.05). A positive relationship between average VC cadences and lower extremity function was also revealed (p < 0.05).

**Conclusion:**

ACT and VC produced similar acute improvements in paretic and non-paretic lower extremity motor function whereas changes in upper extremity motor function were more limited. Faster cycling cadences seem to be associated with greater acute effects.

## 1. Introduction

Post-stroke neuromotor deficits are the leading cause of long-term disability in adults [1]. Arousal of the cortical and subcortical motor areas is a primary mechanism in the recovery of motor function during neurorehabilitation [2]. Exercise is a therapeutic modality which can activate these areas and stimulate the upregulation of trophic and growth factor cascades that ultimately facilitate neuroplasticity and motor recovery [2–5]. Acute effects of exercise can sometimes indicate the efficacy of an exercise intervention. For instance, greater normalized motor evoked potentials have been found following mechanical stimulation of the hand with 25 Hz compared to 10 Hz and the effects of the 25 Hz stimulation appeared to last longer (1-2 hours post-stimulation). In patients with Parkinson's disease, forced lower extremity cycling exercise acutely improved cortical and subcortical activation patterns during a grip force modulation task and 8 weeks of forced lower extremity cycling intervention were also associated with chronic and lasting improvements in grip force modulation [6, 7].

The acute effects of Assisted Cycling Therapy (ACT) with the lower extremities on lower and upper post-stroke motor function have not been investigated. Trials of ACT have produced promising results in persons with Parkinson's disease and persons with Down syndrome [8–11]. During Assisted Cycling Therapy (ACT) an electric motor transmits torque to the pedals of a specialized stationary recumbent bicycle (Theracycle model by Exercycle) to facilitate the pedaling motion of the legs. The arms are not used in this exercise modality. The right and left pedals are interlocked and cannot be moved independently of one another. Usually, this would encourage non-symmetric use of the lower extremities as the pedal stroke of the paretic limb can be completed with the help of the momentum generated by the non-paretic limb. However, during ACT, the rider does not have to rely on the torque contribution of the non-paretic leg. Most of the torque and power output is generated by the motor and a constant cadence is maintained which promotes more symmetric kinematics across both limbs [12]. The motor maintains a pre-programmed cadence regardless of the power contribution by the cyclist. ACT at low cadences (30-50 rpm), often referred to as passive cycling, is sometimes used in the acute phase after a stroke for patients with impaired motor function and insufficient active muscular contractions in the lower extremities for aerobic exercise [12] and ACT may also benefit motor recovery during the chronic post-stroke period based on a case report [13]. However, this case report was limited to one participant and the intervention also included repetitive task practice. Thus, more evidence about the effects of ACT at faster cadences on global (upper and lower extremities) motor function during the chronic post-stroke period is needed. Promisingly, ACT at relatively fast cadences (~80 rpm) has been shown to stimulate blood flow and neural activity bilaterally in the sensorimotor cortices, premotor cortices, and supplemental motor areas to the same degree that active cycling does in persons post-stroke, with the exception of the sensorimotor cortex on the unaffected side [14].

ACT may be particularly useful for people with low cardiorespiratory fitness levels. Maximal aerobic capacities can be reduced by 50% post-stroke [15] and this may limit the ability to sustain a movement rate and duration that optimizes neuroplastic effects and motor recovery [16, 17]. For instance, voluntary cycling at 50 rpm did not increase excitability or neuroplasticity in people with chronic stroke [16]. However, a positive correlation between ACT cadences and changes in functional connectivity between the thalamus and primary motor cortex has been reported in persons with Parkinson's disease (PD) [18]. On average, the assisted cadence was 43% faster than the voluntary cadence and there was no evidence of diminishing returns at assisted cadences up to 95 rpm in regard to functional connectivity.

Thus, ACT may be most beneficial at fast cadences (e.g., 80 rpm) as it is in line with massed practice paradigms [13, 17–22]. ACT at a fast cadence is a way to complete more repetitions in a given amount of time. This is why we examined whether cadence is predictive of changes in motor function in the current study.

In view of limited evidence to date, the primary purpose of this pilot study was to compare the acute effects of ACT, VC (voluntary cycling), and NC (no cycling) on upper and lower, paretic and nonparetic extremity motor function in people during the chronic period after stroke. Based on previous evidence of the effects of ACT on global motor function in other populations [7, 8, 10, 11, 17, 23], we hypothesized that upper and lower extremity motor function would benefit more from ACT than from VC or NC. A secondary purpose was to explore the association of intervention parameters (ratings of perceived exertion [RPE], heart rate, and cadence) and baseline motor function with the amount of change in motor function. To inform clinical practice, it is important to examine extraneous variables which can impact the therapeutic effectiveness of interventions. Sullivan and colleagues [24] found a positive dose-response relationship between exercise intensity and performance on a finger-to-nose task. We therefore used RPE and heart rate, as measures of exercise intensity and as potential predictors of changes in motor function. We hypothesized a positive relationship of RPE and heart rate during cycling with changes in upper and lower extremity motor performance. Based on previous research, we also hypothesized that cadence shares a positive relationship with changes on motor performance [18]. Lastly, we hypothesized that those with better baseline motor function will experience greater acute motor performance benefits and that they would be able to cycle at faster cadences. If both hypotheses were supported, it would indicate that those cycling at faster cadences may not experience benefits only because they cycle faster but because of their better baseline motor function.

## 2. Methods

### 2.1. Participants

**Figure 1:**
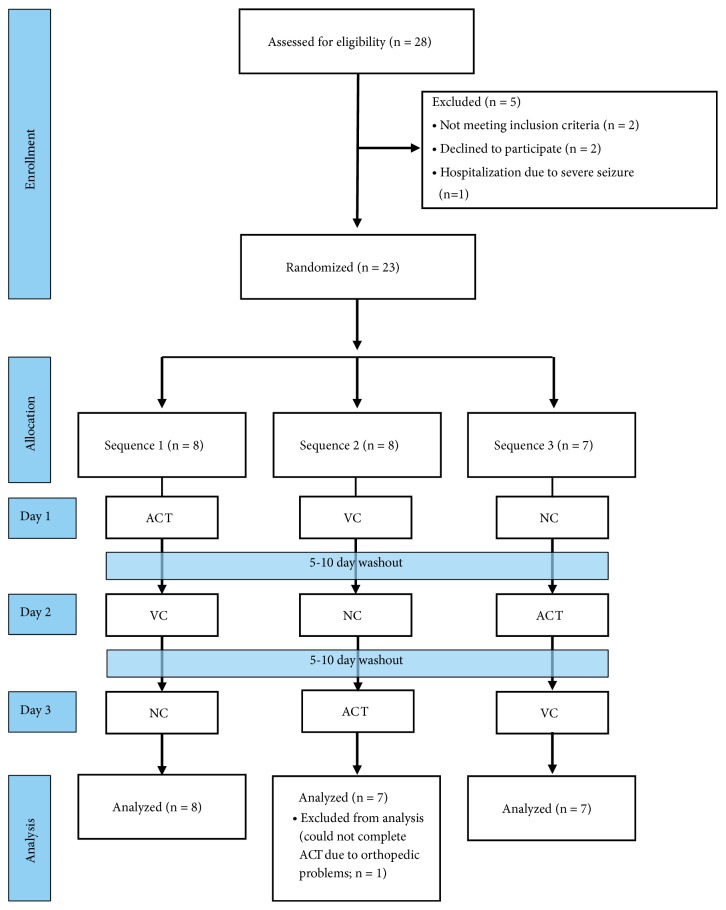
Study flow diagram indicating inclusion, exclusion, and randomization.

Participants were recruited through newspaper ads, from outpatient rehabilitation clinics, and from stroke support groups in the Phoenix metropolitan area. Twenty-two participants completed this study (see Figure 1 for flow-diagram). Participants had suffered at least one unilateral hemorrhagic or ischemic stroke at least six months ago, had residual hemiparesis, were at least 18 years of age, were medically stable, had controlled blood pressure levels (resting blood pressure < 140/90 mmHg), scored at least 24 on the Mini Mental State Exam (MMSE), and scored no higher than three on the Modified Ashworth Scale (MAS). Persons with severe aphasia that precluded comprehension and completion of tests and persons with other neurological conditions were excluded. See Table 1 for participant characteristics.

### 2.2. Design

This was a quasi-counterbalanced cross-over trial. Every participant completed four visits to our research laboratory spaced five to 10 days apart. The first visit consisted of the informed consent process, screening procedures, and the collection of descriptive measures. The following three visits consisted of a session of ACT, a session of VC, or a session of NC. The sequence in which participants completed these sessions was quasi-counterbalanced across participants (see Figure 1). We chose 3 sequences which would allow ACT to be first, second, and third in the sequence and as well as VC and NC to be first second and third. We felt that this degree of counterbalancing was sufficient as we assumed our participants would experience minimal carryover effects. Thus, we used 3 sequences by administering every treatment once across sequence and period. Motor function testing was completed before (i.e., pre-testing) and immediately after each session (i.e., post-testing). Post-testing commenced within five minutes after completion of the given intervention session.

### 2.3. Descriptive Measures

All measures used in this study were administered face-to-face and on a one-on-one basis by a clinically trained researcher. The MMSE [25], Physical Activity Scale for Individuals with Physical Disabilities (PASIPD) [26], Beck Depression Inventory (BDI) [27], Fugl-Meyer Assessment for the lower (LEFMA) and upper extremity (UEFMA) [28], and the Modified Ashworth Scale (MAS) [29] were administered according to standard procedures by a trained clinician. The LEFMA was used as a measure of baseline lower extremity motor function and the UEFMA as a measure of baseline upper extremity motor function.

### 2.4. Outcome Measures

Paretic and non-paretic upper extremity motor performance was assessed during pre- and post-testing with the Box and Blocks Test (BBT) [30–32]. The BBT was chosen due to its good criterion validity and its wide use in research and clinical practice which makes the results of the current study easily understandable and interpretable [30, 31]. This test required participants to move wooden cubes (16.39 cm^3^) from one box (25.4 cm x 25.4 cm) to another box of equal size across a barrier that was 15.2 cm in height. The test was administered according to standard procedures [33].

Lower extremity motor performance was tested with the Lower Extremity Motor Coordination (LEMOCOT) test. The test was administered in accordance with standardized procedures [34]. It was completed in a seated position and required participants to alternately touch two red dots with their big toe on a board that was placed on the floor in front of the participant. The dots were spaced 30 cm apart and arranged proximally and distally on the intersecting line of the sagittal and transverse plane in front of the participant with the proximal dot placed directly under the participant's heel when the knee was flexed to 90 degrees. Participants were required to alternately touch the dots with their big toe as fast as possible. This task required cyclical knee extension and flexion, slight ankle plantar and dorsi flexion, and very slight hip flexion and extension, similar to the musculoskeletal requirements of cycling. Participants completed a 5-10-second practice trial and then three 20-second test trials with a one-minute break between trials. This was first completed with the non-paretic leg and then with the paretic leg. The average of the second and third trials was used as the outcome measure [35].

### 2.5. Interventions

All cycling sessions lasted 25 minutes and were completed on a stationary, recumbent research prototype cycle ergometer (Theracycle) that was built by the Exercycle Company (Franklin, MA). The electric motor that was built into the bicycle could turn the pedals at up to 95 rpm regardless of the power contribution by the participant. The pedals were specialized platform pedals with metal cuppings and Velcro straps that prevented the feet from slipping off the pedals in any direction. For a thorough description of the bike, see Ringenbach et al. [36, 37]

ACT sessions began with a five-minute voluntary warm-up without the help of the motor. We aimed for the target ACT cadence to be 1.8 times greater than the average warm-up cadence as previous research with persons with DS (Down syndrome) has shown that an ACT cadence which is 80% greater than the voluntary cadence may be beneficial for motor perfrmance and cognitive function [11, 36, 37]. However, the minimum target cadence was 80 rpm because an assisted cadence of at least 80 rpm has been shown to be beneficial for clinical, motor, and cognitive function of persons with PD [7, 23, 38, 39] and persons with DS [8, 9, 11, 36, 37]. The only previous study of ACT in single participant with stroke utilized a cadence of 80 rpm which was gradually reached after 12 sessions and a starting cadence of 70 rpm [13]. After the five-minute warm up, the motor was turned on and the cadence was set to the average of the warm-up and the target cadence for 5 minutes to allow participants to become familiar with ACT. Subsequently, the motor was programmed to maintain the target cadence for 15 minutes. If participants were uncomfortable at the prescribed target cadence, then the cadence was lowered in 5 rpm increments until the participant felt comfortable. Participants were not encouraged to pedal faster than the target cadence.

For VC sessions, participants were instructed to complete a five-minute warm-up by cycling at their own preferred cadence and then to continue cycling for 20 minutes at their preferred cadence. The motor was not turned on and participants were not encouraged to pedal faster or slower at any point. The resistance that participants were cycling against was 0.5 kp.

During NC sessions, participants also sat on the bicycle with their feet strapped into the pedals but they did not cycle. During the 25-minute NC session, participants engaged in a conversation about their physical activity habits with the researcher which always concluded by the researcher informing the participant of the post-stroke physical activity recommendations [40].

### 2.6. Statistical Analyses

The outcome measures were converted into change scores by subtracting the pre-test scores from the post-test scores. Thus, a positive change score indicates an improvement. Numerous change scores were not normally distributed depending on the condition as indicated by Shapiro-Wilk tests. Therefore, all change scores were transformed as an inverse unit (1/x). First, all values were multiplied by -1 and then 1 was added to all values before inversion as there cannot be a 0 in the denominator. Once the transformation was completed the ordering of the data was identical to the original data. The transformed change scores were normally distributed within each intervention as verified with Shapiro-Wilk tests.

Linear mixed model (LMM) analyses were used to test the main effects of sequence and intervention. Tukey's HSD post hoc analyses were used to test differences among the change scores of the three interventions. The models were computed with and without the following covariates: BDI, months since stroke, and MAS scores. In addition, LEFMA scores were entered as a covariate for the LEMOCOT change scores and UEFMA was entered for the BBT change scores. Caffeine consumption before the lab visit and months since stroke was also included as covariates. Caffeine consumption was included as a covariate because it has been shown to benefit gross motor performance and potentially impair fine motor performance [41, 42]. To test within-intervention effects, paired samples t-tests were completed with pre- and post-tests scores, separately for each intervention.

Next, linear ordinary least squares (OLS) regression models were computed to analyze the associations of RPE, percent of heart rate reserve (%HRR), cycling cadence, LEFMA, and UEFMA with change scores in motor outcome measures, in order to examine the effects of rate of movement, exercise intensity, and baseline motor function on the degree of change in motor measures. These linear trends were analyzed separately for each intervention. All *β* values listed are unstandardized. We also computed Pearson r correlation coefficients between LEFMA scores and ACT and VC cadences to assess the relationship between baseline motor function and cycling cadence. Analyses were completed with SPSS v. 22. Two-tailed type I error probability was set at *α* = 0.05.

## 3. Results

### 3.1. Treatment Fidelity

No adverse events occurred during or as a result of the interventions. All 22 participants completed all three intervention sessions and no session was terminated prematurely. Intervention parameters are summarized in Table 2. The mean ACT cadence was 83.8 ± 23.9% (mean ± SD) faster than the voluntary warm-up cadence. However, three participants were not comfortable cycling the minimum prescribed 80 rpm. Their maximum cadences during ACT were 66 rpm, 70 rpm, and 74 rpm, which was still at least 73% faster than their VC cadence. The mean VC cadence was significantly slower than the mean ACT cadence, but the heart rates did not differ between ACT and VC (see Table 2).

### 3.2. Main and Intervention Effects

The LMM results did not differ whether covariates were included or not, and none of the covariates were significant. Thus, we are only reporting the statistics from the models without covariates. There was no main effect of sequence indicating that there were no carryover effects. Analyses yielded a significant intervention effect for the LEMOCOT on the paretic side (F(2,41) = 3.74; p = 0.036) and the non-paretic side (F(2,41) = 16.42; p < 0.001) and for the BBT on the non-paretic side (F(2,41) = 11.13; p < 0.001). The post hoc analyses showed that the change score for the LEMOCOT on the paretic side was greater for ACT compared to NC, but VC did not differ significantly from ACT or NC. Changes score for the LEMOCOT on the non-paretic side were greater for ACT and VC compared to NC. Finally, changes score for the BBT on the non-paretic side were greater for ACT and VC compared to NC (see Table 3). For each test (LEMOCOT and BBT), there were six participants who not could execute a single successful toe-touch or block transfer during pre- and post-testing. Thus, their pre- and post-test scores were zero for those tests. We eliminated the data of these participants and reran the LMM, post hoc, and paired samples t-test analyses. The results did not change: there were significant intervention effects for LEMOCOT-P (F(2,29) = 4.17; p = 0.026), LEMOCOT-NP (F(2,29) = 16.08; p < 0.001), and BBT-NP (F(2,29) = 7.64; p = 0.002), but not for BBT-P (F(2,29) = 0.87; p = 0.430). For the sake of brevity, we will not list the post hoc and paired-sample t-test results here, as they were identical to the post hoc and paired-sample t-test results from the full data set. Pre- and posttest means and standard deviations for each test by intervention as well as paired sample t-test results are listed in Table 3.

### 3.3. Trend Analyses

**Figure 2:**
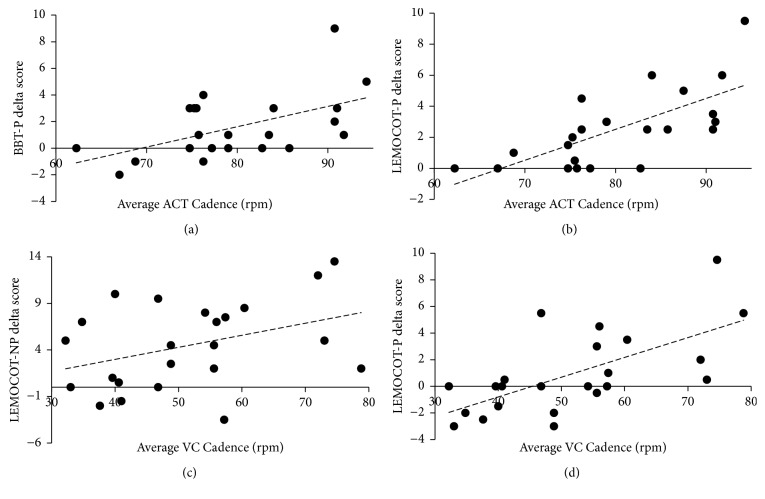
Significant positive linear trends (p < 0.05) between (a) BBT-P delta scores and cadence during ACT; (b) LEMOCOT-P delta scores and cadence during ACT; (c) LEMOCOT-NP delta scores and cadence during VC; (d) LEMOCOT-P delta scores and cadence during VC. Note: A positive delta score indicates improvement. The average ACT cadence is a weighted average of the first 5 minutes at a lower ACT cadence and the following 15 minutes at the ACT target cadence. ACT = Assisted Cycling Therapy; BBT-NP = Box and Blocks Test-non-paretic; BBT-P = Box and Blocks Test-paretic; LEMOCOT-NP = Lower Extremity Motor Coordination Test-non-paretic; LEMOCOT-P = Lower Extremity Motor Coordination Test-paretic; VC = voluntary cycling.

The trend analyses indicated the following for the ACT intervention. A negative linear trend was found for RPE and BBT-NP change scores (F(1,19) = 6.01; p < 0.05; R^2^ = 0.23; *β* = -0.91). A positive linear trend was found for cadence and LEMOCOT-P (F(1,19) = 10.54; p < 0.05; R^2^ = 0.36; *β* = 0.19) as well as BBT-P (F(1,19) = 5.91; p < 0.05; R^2^ = 0.16; *β* = 0.16). Regarding the VC intervention, the trend analyses revealed a negative linear association between RPE and LEMOCOT-P (F(1,19) = 5.66; p < 0.05; R^2^ = 0.26; *β* = -6.30) and a positive linear association for cadence and LEMOCOT-P (F(1,19) = 16.02; p < 0.001; R^2^ = 0.45; *β* = 0.16) and for cadence and LEMOCOT-NP (F(1,19) = 4.64; p < 0.05; R^2^ = 0.17; *β* = 0.16). See Figure 2 for the plots of the significant trends between cadence and change scores. No significant trend was found for %HRR during ACT or VC and motor measures. No significant trend was found for LEFMA scores and LEMOCOT-P change scores for ACT or VC. A positive linear trend emerged for UEFMA scores and BBT-P change scores for both ACT (F(1,19) = 6.96; p < 0.05; R^2^ = 0.26; *β* = 0.07) and VC (F(1,19) = 5.37; p < 0.05; R^2^ = 0.20; *β* = 0.08). LEFMA scores did not correlate significantly with VC cadences (r = 0.293; p = 0.175) or ACT cadences (r = 0.126; p = 0.575).

## 4. Discussion

### 4.1. Intervention Characteristics and Main Effects of the Interventions

The mean ACT cadence was 79.5 rpm and the mean VC cadence was 51.5. Heart rates, %HRR, and RPE did not differ between ACT and VC, but these measures were significantly lower during NC (see Table 2). Thus, the only apparent difference between ACT and VC was the rate of movement whereas the cardiorespiratory and perceived exercise intensities did not differ.

The results only partially support our hypothesis of greater increases in BBT and LEMOCOT scores following ACT compared to VC or NC. The main intervention effects indicated a significant difference between the interventions for all assessments except for the BBT-P. The post hoc analyses did not indicate a difference in lower or upper extremity paretic or non-paretic motor performance between ACT and VC. However, the change scores for ACT and VC were typically greater than the change scores for NC, with the exception that the change scores between VC and NC did not differ for the LEMOCOT-P. Within-intervention analyses indicated a significant positive effect of ACT on all assessments. The only other significant pre- to posttest improvements were found for VC on the non-paretic lower-extremity (LEMOCOT-NP) and NC on the non-paretic upper extremity (BBT-NP). Thus, our results seem to show more salient effects of ACT compared to VC or NC on upper and lower extremity non-paretic and paretic motor performance. The improvement in function of the paretic upper extremity following lower extremity exercise is consistent with other work [43].

Other studies have also found ACT to be more effective than VC in regard to the motor control of persons with PD [7, 23, 38, 39] or persons with DS [8, 9, 11, 36, 37]. It should be noted that heart rates do not differ between ACT and VC regardless of study, including this one. It has been hypothesized that the benefits of ACT stem from the faster than voluntary cycling cadence and associated augmented afferent corticospinal stimulation rather than the cardiovascular stress or arousal [7, 17, 18, 23, 44, 45]. It is also plausible that the greater number of revolutions during ACT created a practice effect that could in part be responsible for the observed changes [21, 22].

### 4.2. Relationships between Intervention Parameters and Acute Response

The results of the present study partially support our hypothesis of the positive relationships between cadence and motor performance. We found positive linear relationships between the ACT cadence and changes in the paretic lower and upper extremity motor performance. We also found a positive relationship between the VC cadence and changes in paretic and non-paretic lower extremity performance. However, it appears that cadences close to or over 80 rpm are necessary for changes in paretic upper extremity motor performance to occur. This is consistent with the lack of effect of cycling at 50 rpm on post-stroke motor cortex excitability and neuroplasticity [16]. The relationship of ACT but not VC cadence with paretic upper extremity performance indicates that faster than voluntary cadences may be necessary for benefits in non-task specific or global motor performance changes.

Contrary to our hypothesis, a significant negative linear association was present between RPE and change in BBT-NP for the ACT intervention and between RPE and change in LEMOCOT-P for the VC intervention. These trends indicate that higher levels of perceived exertion may not be beneficial for acute motor performance changes. This is in accordance with a negative relationship between perception of effort and central motor drive in persons with chronic fatigue syndrome [46]. Relatively high RPE during exercise can lead to central fatigue [47–50] which in turn may have compromised cortical and sub-cortical output [46, 51]. Locomotor activities are thought to tax a large portion of the computational capacity of reticular formations, subcortical, and cortical areas [47]. This demand could be exacerbated in persons with stroke-induced hemiparesis because of difficulties with paretic extremity control [50].

The hypothesis of positive associations between baseline motor function and degree of change in motor performance is only partially supported. We found no relationships between baseline lower extremity function and changes in lower extremity motor performance following ACT or VC. But, we did find positive linear trends between baseline upper extremity motor function and changes in upper extremity motor performance. Thus, the changes in lower extremity motor performance are more likely attributable to the rate of movement during cycling rather than lower extremity motor function. This is supported by the lack of correlation of baseline lower extremity motor function with ACT or VC cycling cadences. This latter finding was contrary to our hypothesis of a positive relationship between baseline motor function and cycling cadence.

We found significant improvements in motor performance of the paretic upper extremity following ACT. As mentioned, these improvements are positively related to the ACT cycling cadence and they are positively related to the baseline upper extremity motor function. However, there is no theoretical basis for a relationship between baseline upper extremity motor function and lower extremity cycling cadence. Thus, ACT cycling cadence and baseline upper extremity motor function may have contributed independently to the observed improvements in upper extremity motor performance. The assumptions of a mediating role of cycling cadence between baseline upper extremity motor function and post-exercises changes in upper extremity motor performance are violated. The violation of a mediation is due to the lack of relationship, either theoretical or statistical, between the independent variable (baseline upper or lower motor function, respectively) and the mediating variable (cycling cadence). Thus, baseline upper extremity motor function and cycling cadence likely exerted independent effects on acute changes in motor performance. The influence of baseline upper extremity motor function on changes in upper extremity motor performance following cycling also appeared in the VC intervention, where baseline UEFMA and change in BBT-P shared a positive trend. However, the change in BBT-P following VC was not significant which again indicates that the VC cadences were overall not sufficient to elicit a positive change in motor performance. However, the influence of baseline motor function on changes in upper and lower extremity motor performance appears to be limited because the UEFMA and LEFMA scores were entered as covariates in the LMM (main effects) analyses but they did not explain a significant amount of variance in the LEMOCOT and BBT change scores, respectively.

It is important to point out that the observed trends between cadence and motor performance changes as well as RPE and motor performance do not represent dose-response relationships, as the dose was not manipulated but rather selected based on participant characteristics, such a tolerance, and then held constant. Thus, the cycling cadences during ACT were influenced by the participants' preferences. The question of whether forcing a faster ACT or VC cadence on the participants would have conferred greater benefit remains to be answered. It appears that ACT may be more effective than VC in increasing motor performance acutely but maybe this is only the case as long as the ACT cadence does not exceed a certain tolerance limit. The results of our study seem to imply that ACT cadence shares a positive relationship with acute changes in motor performance, as long as the ACT cadence is influenced by the voluntary cycling cadence and does not exceed a certain comfort level. Future research needs to investigate whether an “involuntary” increase in ACT cadence increases the acute benefits for those who would otherwise cycle too slowly to experience positive performance changes.

### 4.3. Limitations

The BBT and LEMOCOT tests both suffered from a floor effect. For each test there were six participants who could not complete even one successful block transfer or toe touch with the paretic limb either on the pre- or on posttest. Thus, these tests may have been unable to detect changes in participants with very poor motor function. Tests that can detect very small changes, such as range of motion tests, should be incorporated in future studies. However, the significant pre- to post-changes speak for the efficacy of the intervention despite six participants who experienced no detectable change.

### 4.4. Conclusion

ACT was feasible and safe for all of our participants and the mean heart rates stayed around 90 bpm. Thus, ACT may not be overly fatiguing and allow for occupational and physical therapy sessions to take place afterwards. However, other measures of stress and exertion should be monitored (e.g., RPE) because they may be associated with negative effects on motor performance. It does appear that faster cadences are associated with greater acute motor performance benefits and ACT seems to be an effective way of accomplishing relatively high cadences without vigorous exertion. The cadences achieved during VC were about 28 rpm lower at similar heart rates and RPE values.

ACT and VC resulted in similar acute increases in motor performance, although the changes appear a little larger following ACT. The acute benefits in motor performance following ACT with the lower extremities appear to be global as the upper extremities also benefited even though they were not involved in the exercise.

Future studies should investigate whether ACT at fast cycling cadences increases motor cortex excitability similarly to transcranial magnetic stimulation and whether ipsilesional or contralesional excitability is associated with improvement in motor performance on the paretic side. It should also be investigated whether cortical excitability and changes in motor performance are associated with power output by the paretic and non-paretic leg. This could be measured via force plates in the pedals or bilateral power meters. To shed more light on interindividual variability of motor recovery in response to ACT or VC, future research should investigate the effect of leg cycling on contralateral sensorimotor cortex activation. The degree of activity of the contralateral sensorimotor cortex in response to passive movement may be predictive of motor recovery [52].

## Figures and Tables

**Table 1:** Descriptive statistics.

Gender (m/f)	16/6
Lesion side (r/l)	7/15
Type of stroke	
Ischemic (n)	12
Hemorrhage (n)	10
Age (years; mean ± SD)	60.26 ± 15.55
MSS (mean ± SD)	95.70 ± 85.26
Assistive device including AFOs (n)	16
Aphasia (n)	6
BB medication (n)	16
BMI (kg/m^2^; mean ± SD)	30.17 ± 6.18
MMSE (mean ± SD)	26.90 ± 3.09
BDI (mean ± SD)	8.82 ± 5.26
PASIPD (mean ± SD)	37.5 ± 21.16
LEFMA (mean ± SD)	21.00 ± 8.01
UEFMA (mean ± SD)	34.63 ± 18.15

*Note.* AFO = ankle foot orthosis; BB = beta blocker; BDI = Beck Depression Inventory; BMI = body mass index; LEFMA = Lower Extremity Fugl-Meyer Assessment; MMSE = Mini Mental State Examination; MSS = Months Since Stroke; PASIPD = Physical Activity Scale for Individuals with Physical Disabilities; UEFMA = Upper Extremity Fugl-Meyer Assessment.

**Table 2:** Mean differences in RPE, HR, %HRR, and CAD across interventions.

	ACT	VC	NC		p	Post-hoc comparisons
RPE	11.9 ± 2.0	11.2 ± 1.7	7.5 ± 1.3	F(2,64) = 46.24	< 0.001	ACT, VC > NC
HR	90.3 ± 17.5	92.3 ± 21.3	74.3 ± 15.0	F(2,64) = 6.79	0.002	ACT, VC > NC
%HRR	27.8 ± 17.3	31.8 ± 25.2	4.8 ± 2.5	F(2,64) = 14.77	< 0.001	ACT, VC > NC
CAD	79.5 ± 8.5	51.5 ± 13.7		t(21) = 13.96	< 0.001	ACT > VC

*Note.* %HRR = percentage of heart rate reserve; ACT = Assisted Cycling Therapy; CAD = cadence; HR = heart rate; NC = no cycling; RPE = rating of perceived exertion; VC = voluntary cycling.

Values are expressed as mean ± SD.

**Table 3:** Means ± standard deviation for pre- and post-tests in each intervention.

	ACT		VC	
	pre	post	pre	post
LEMOCOT-P	16.47 ± 15.86	18.64 ± 17.11*∗∗*	18.76 ± 17.03	19.67 ± 17.96
LEMOCOT-NP	45.61 ± 11.53	50.70 ± 11.72*∗∗*	45.87 ± 10.73	50.35 ± 10.73*∗∗*
BBT-P	16.77 ± 20.79	18.32 ± 22.34*∗*	18.39 ± 21.82	18.83 ± 22.92
BBT-NP	56.68 ± 10.14	60.41 ± 10.62*∗∗*	57.83 ± 12.26	59.30 ± 12.30

	NC			
	pre	post		

LEMOCOT-P	18.93 ± 17.45	19.26 ± 18.18		
LEMOCOT-NP	49.67 ± 12.62	47.93 ± 12.11		
BBT-P	19.74 ± 22.85	20.17 ± 22.65		
BBT-NP	60.57 ± 12.66	58.52 ± 11.97*∗*		

*Note.* ACT = Assisted Cycling Therapy; BBT-NP (Box and Blocks Test-non-paretic): number of successfully transported blocks in minute with the non-paretic arm; BBT-P (Box and Blocks Test-paretic): number of successfully transported blocks in 1 minute with the paretic arm; LEMOCOT-NP (Lower Extremity Motor Coordination Test-non-paretic): mean number of successful toe-touches in 20 seconds with the non-paretic leg; LEMOCOT-P (Lower Extremity Motor Coordination Test - paretic): mean number of successful toe-touches in 20 seconds with the paretic leg.

Differences between pre- and posttest means were tested with paired samples t-tests (df = 21): *∗*p < 0.05, *∗∗*p < 0.001.

## Data Availability

The data used to support the findings of this study are available from the corresponding author upon request.

## References

[1] Dobkin B. H. (2005). Rehabilitation after stroke. *The New England Journal of Medicine*.

[2] Goldfine A. M., Schiff N. D. (2011). What is the role of brain mechanisms underlying arousal in recovery of motor function after structural brain injuries?. *Current Opinion in Neurology*.

[3] Knaepen K., Goekint M., Heyman E. M., Meeusen R. (2010). Neuroplasticity exercise-induced response of peripheral brain-derived neurotrophic factor: A systematic review of experimental studies in human subjects. *Sports Medicine*.

[4] Mang C. S., Campbell K. L., Ross C. J. D., Boyd L. A. (2013). Promoting neuroplasticity for motor rehabilitation after stroke: Considering the effects of aerobic exercise and genetic variation on brain-derived neurotrophic factor. *Physical Therapy in Sport*.

[5] Piepmeier A. T., Etnier J. L. (2015). Brain-derived neurotrophic factor (BDNF) as a potential mechanism of the effects of acute exercise on cognitive performance. *Journal of Sport and Health Science*.

[6] Christova M., Rafolt D., Golaszewski S., Gallasch E. (2011). Outlasting corticomotor excitability changes induced by 25 Hz whole-hand mechanical stimulation. *European Journal of Applied Physiology*.

[7] Alberts J. L., Linder S. M., Penko A. L. (2011). It is not about the bike, it is about the pedaling: forced exercise and Parkinson’s disease. *Exercise and Sport Sciences Reviews*.

[8] Holzapfel S. D., Ringenbach S. D. R., Mulvey G. M. (2015). Improvements in manual dexterity relate to improvements in cognitive planning after assisted cycling therapy (ACT) in adolescents with down syndrome. *Research in Developmental Disabilities*.

[9] Holzapfel S. D., Ringenbach S. D. R., Ganger R. O. (2016). Older adults with down syndrome benefit from assisted cycling therapy: Implications for physical activity, fitness, and daily function. *Compilation of Recent Research/Review Works in the Field of Down Syndrome*.

[10] Ridgel A. L., Peacock C. A., Fickes E. J., Kim C. (2012). Active-assisted cycling improves tremor and bradykinesia in parkinson’s disease. *Archives of Physical Medicine and Rehabilitation*.

[11] Ringenbach S. D. R., Albert A. R., Chen C.-C., Alberts J. L. (2014). Acute bouts of assisted cycling improves cognitive and upper extremity movement functions in adolescents with down syndrome. *The American Journal on Intellectual and Developmental Disabilities*.

[12] Ferrante S., Ambrosini E., Ravelli P. (2011). A biofeedback cycling training to improve locomotion: A case series study based on gait pattern classification of 153 chronic stroke patients. *Journal of NeuroEngineering and Rehabilitation*.

[13] Linder S. M., Rosenfeldt A. B., Rasanow M., Alberts J. L. (2015). Forced aerobic exercise enhances motor recovery after stroke: A case report. *American Journal of Occupational Therapy*.

[14] Lin P.-Y., Chen J.-J. J., Lin S.-I. (2013). The cortical control of cycling exercise in stroke patients: An fNIRS study. *Human Brain Mapping*.

[15] Kelly J. O., Kilbreath S. L., Davis G. M. (2003). Cardiorespiratory fitness and walking ability in subacute stroke patients. *Archives of Physical Medicine and Rehabilitation*.

[16] Murdoch K., Buckley J. D., McDonnell M. N. (2016). The effect of aerobic exercise on neuroplasticity within the motor cortex following stroke. *PLoS ONE*.

[17] Shah C., Beall E. B., Frankemolle A. M. M. (2016). Exercise therapy for parkinson’s disease: Pedaling rate is related to changes in motor connectivity. *Brain Connectivity*.

[18] Beall E. B., Lowe M. J., Alberts J. L. (2013). The effect of forced-exercise therapy for parkinson’s disease on motor cortex functional connectivity. *Brain Connectivity*.

[19] Lang C. E., MacDonald J. R., Gnip C. (2007). Counting repetitions: An observational study of outpatient therapy for people with hemiparesis post-stroke. *Journal of Neurologic Physical Therapy*.

[20] Mark V. W., Taub E. (2004). Constraint-induced movement therapy for chronic stroke hemiparesis and other disabilities. *Restorative Neurology and Neuroscience*.

[21] Sterr A., Elbert T., Berthold I. (2002). Longer versus shorter daily constraint-induced movement therapy of chronic hemiparesis: An exploratory study. *Archives of Physical Medicine and Rehabilitation*.

[22] Cooke E. V., Mares K., Clark A., Tallis R. C., Pomeroy V. M. (2010). The effects of increased dose of exercise-based therapies to enhance motor recovery after stroke: A systematic review and meta-analysis. *BMC Medicine*.

[23] Ridgel A. L., Vitek J. L., Alberts J. L. (2009). Forced, not voluntary, exercise improves motor function in Parkinson’s disease patients. *Neurorehabilitation and Neural Repair*.

[24] Sullivan S. J., Schneiders A. G., Handcock P. (2011). Changes in the timed finger-to-nose task performance following exercise of different intensities. *British Journal of Sports Medicine*.

[25] Tombaugh T. N., McIntyre N. J. (1992). The mini-men tal state exam ination: A comprehensive review. *Journal of the American Geriatrics Society*.

[26] Washburn R. A., Zhu W., McAuley E., Frogley M., Figoni S. F. (2002). The physical activity scale for individuals with physical disabilities: Development and evaluation. *Archives of Physical Medicine and Rehabilitation*.

[27] Beck A. T., Steer R. A., Garbin M. G. (1988). Psychometric properties of the beck depression inventory: Twenty-five years of evaluation. *Clinical Psychology Review*.

[28] Fugl-Meyer A. R., Jääskö L., Leyman I. (1975). The post-stroke hemiplegic patient. 1. a method for evaluation of physical performance. *Journal of rehabilitation medicine*.

[29] Gregson J. M., Leathley M., Moore A. P. (1999). Reliability of the tone assessment scale and the modified Ashworth scale as clinical tools for assessing poststroke spasticity. *Archives of Physical Medicine and Rehabilitation*.

[30] Chen H.-M., Chen C. C., Hsueh I.-P. (2009). Test-retest reproducibility and smallest real difference of 5 hand function tests in patients with stroke. *Neurorehabilitation and Neural Repair*.

[31] Lin K., Chuang L., Wu C. (2010). Responsiveness and validity of three dexterous function measures in stroke rehabilitation. *Journal of Rehabilitation Research & Development*.

[32] Platz T., Pinkowski C., van Wijck F. (2005). Reliability and validity of arm function assessment with standardized guidelines for the Fugl-Meyer test, action research arm test and box and block test: A multicentre study. *Clinical Rehabilitation*.

[33] Mathiowetz V., Volland G., Kashman N., Weber K. (1985). Adult norms for the box and block test of manual dexterity. *The American Journal of Occupational Therapy*.

[34] Desrosiers J., Rochette A., Corriveau H. (2005). Validation of a new lower-extremity motor coordination test. *Archives of Physical Medicine and Rehabilitation*.

[35] Pinheiro M. B., Scianni A. A., Ada L. (2014). Reference values and psychometric properties of the lower extremity motor coordination test. *Archives of Physical Medicine and Rehabilitation*.

[36] Holzapfel S. D., Ringenbach S. D. R., Mulvey G. M. (2016). Differential effects of assisted cycling therapy on short-term and working memory of adolescents with Down syndrome. *Journal of Cognitive Psychology*.

[37] Ringenbach S. D. R., Holzapfel S. D., Mulvey G. M. (2016). The effects of assisted cycling therapy (ACT) and voluntary cycling on reaction time and measures of executive function in adolescents with down syndrome. *Journal of Intellectual Disability Research*.

[38] Ridgel A. L., Kim C.-H., Fickes E. J. (2011). Changes in executive function after acute bouts of passive cycling in parkinson’s disease. *Journal of Aging and Physical Activity*.

[39] Ridgel A. L., Phillips R. S., Walter B. L. (2015). Dynamic high-cadence cycling improves motor symptoms in parkinson’s disease. *Frontiers in Neurology*.

[40] Gordon N. F., Gulanick M., Costa F. (2004). Physical activity and exercise recommendations for stroke survivors: An american heart association scientific statement from the council on clinical cardiology, subcommittee on exercise, cardiac rehabilitation, and prevention; the council on cardiovascular nursing; the council on nutrition, physical activity, and metabolism; and the stroke council. *Stroke*.

[41] Smith A. (2002). Effects of caffeine on human behavior. *Food and Chemical Toxicology*.

[42] Spriet L. L. (2014). Exercise and sport performance with low doses of caffeine. *Sports Medicine*.

[43] Ploughman M., McCarthy J., Bossé M. (2008). Does treadmill exercise improve performance of cognitive or upper-extremity tasks in people with chronic stroke? A randomized cross-over trial. *Archives of Physical Medicine and Rehabilitation*.

[44] Christensen L. O. D., Johannsen P., Sinkjaer T. (2000). Cerebral activation during bicycle movements in man. *Experimental Brain Research*.

[45] Alberts J. L., Phillips M., Lowe M. J. (2016). Cortical and motor responses to acute forced exercise in Parkinson’s disease. *Parkinsonism & Related Disorders*.

[46] Sacco R. L., Kasner S. E., Broderick J. P. (2013). An updated definition of stroke for the 21st century: A statement for healthcare professionals from the American heart association/American stroke association. *Stroke*.

[47] Dietrich A., Audiffren M. (2011). The reticular-activating hypofrontality (RAH) model of acute exercise. *Neuroscience & Biobehavioral Reviews*.

[48] MacKay-Lyons M. J., Makrides L. (2002). Exercise capacity early after stroke. *Archives of Physical Medicine and Rehabilitation*.

[49] Michael K., Macko R. F. (2007). Ambulatory activity intensity profiles, fitness, and fatigue in chronic stroke. *Topics in Stroke Rehabilitation*.

[50] Riley N. A., Bilodeau M. (2002). Changes in upper limb joint torque patterns and EMG signals with fatigue following a stroke. *Disability and Rehabilitation*.

[51] Benwell N. M., Sacco P., Hammond G. R. (2006). Short-interval cortical inhibition and corticomotor excitability with fatiguing hand exercise: A central adaptation to fatigue?. *Experimental Brain Research*.

[52] Jang S. H., Kim Y.-H., Chang Y. (2004). The predictive value of cortical activation by passive movement for motor recovery in stroke patients. *Restorative Neurology and Neuroscience*.

